# The Protective Effect of Taurine on Oxidized Fish-Oil-Induced Liver Oxidative Stress and Intestinal Barrier-Function Impairment in Juvenile *Ictalurus punctatus*

**DOI:** 10.3390/antiox10111690

**Published:** 2021-10-26

**Authors:** Yong Shi, Yi Hu, Ziqin Wang, Jiancheng Zhou, Junzhi Zhang, Huan Zhong, Guihong Fu, Lei Zhong

**Affiliations:** 1 Hunan Research Center of Engineering Technology for Utilization of Distinctive Aquatic Resource, Hunan Agricultural University, Changsha 410128, China; shiyong@stu.hunau.edu.cn (Y.S.); huyi0231@hunau.edu.cn (Y.H.); wangziqin202109@163.com (Z.W.); zhjun123@hunau.edu.cn (J.Z.); zhonghuan@hunau.edu.cn (H.Z.); fuguihong@hunau.edu.cn (G.F.); 2 College of Animal Science and Technology, Hunan Agricultural University, Changsha 410128, China; 3 Wuhan Dabeinong Aquatic Science and Technology Co. Ltd., Wuhan 430000, China; jiancheng1949@163.com

**Keywords:** channel catfish, oxidative damage, immune response, intestinal health, signaling pathway

## Abstract

Dietary lipids provide energy for growth and development and provide fatty acids necessary for normal structure and biological function. However, oxidized lipids cause oxidative stress and intestinal damage. An 8-week feeding trial with fresh fish oil (FFO, control group), oxidized fish oil (OFO), and taurine-supplemented diets (OFOT, OFO + 0.2% of taurine) was conducted to evaluate the protective effect of taurine on oxidized fish-oil-induced liver oxidative stress and intestine impairment in juvenile *Ictalurus* *punctatus*. The results showed that (1) Growth performance was significantly lower in fish fed OFO than in those fed other diets, whereas the opposite occurred in the hepatosomatic index. (2) OFO-feeding significantly increased lipid deposition compared with the FFO group. The addition of taurine ameliorated the OFO-induced increase in lipid vacuolization in the liver, significantly upregulated *lpl* mRNA expression, and downregulated *fas* and *srebp1* mRNA expression. (3) OFO-feeding significantly reduced oxidative damage of liver. Compared with the OFO group, the OFOT group remarkably upregulated antioxidant enzyme mRNA expression through the Nrf2-Keap1 signaling pathway based on the transcriptional expression. (4) OFO diets induced intestinal physical and immune barrier damage. Compared with the OFO group, OFOT diets remarkably downregulated *il-1β*, *il-6*, *tnf-α*, and *il-8* mRNA expression and upregulated *tgf-β* mRNA expression through the NF-κB signaling pathway. Besides, the addition of taurine to OFO diets significantly upregulated *zo-2* and *zo-1* mRNA expression, and downregulated *claudin-15* and *claudin-12* mRNA expression. In conclusion, oxidized-fish-oil diets can cause negative physiological health effects in *Ictalurus* *punctatus*, while adding taurine can increase growth and antioxidant ability, reduce lipid deposition, and improve intestinal health.

## 1. Introduction

It is well known that, as one of the important nutrients of aquatic animals, dietary lipids not only provide energy for growth and development in fish, but also provide the essential fatty-acid and fat-soluble vitamins that maintain normal structure and biological function [1]. At present, the main lipid sources in aquatic feed are fish oil and soybean oil. Compared with soybean oil, fish oil has a high content of unsaturated fatty acids (HUFAs), such as eicosapentaenoic acid (EPA) and docosahexaenoic acid (DHA), and a good feeding attraction effect, so it is the best lipid source for aquatic animals [2,3]. However, EPA and DHA are easily oxidized during the storage and processing of fish oil and feed, producing harmful substances such as lipid hydroperoxides, ketones, aldehydes, and acids [4,5]. Studies have also reported that feeding an oxidized-fish-oil diet has been found to decrease growth performance and cause oxidative stress of tilapia (*Oreochromis niloticus*) [6], Wuchang bream (*Megalobrama amblycephala*) [7], and orange spotted grouper (*Epinephelus coioides*) [5], disrupt lipid metabolism of *Rhynchocypris lagowski* Dybowski [8], and induce intestinal injury of *Megalobrama amblycephala* [9] and rice field eel (*Monopterus albus*) [10]. Therefore, exploring effective dietary strategies is imperative to alleviate the negative effects of oxidized-fish-oil diets on aquatic animals.

Taurine is a type of non-protein amino acid in the form of a free amino acid, which has a wide range of physiological functions, such as calcium homeostasis, osmotic regulation, membrane stability, and antioxidant and anti-inflammatory functions [11,12]. Many studies have also demonstrated the versatility of taurine in aquatic animals. Previous studies in our laboratory indicated that taurine supplementation in a low-fish-meal diet increased growth performance and immunity function and enhanced anti-stress ability in black carp (*Mylopharyngodon piceus*) [13] and rice field eel [14]. Similar studies have found, in other aquatic animals, that taurine can increase growth performance, enhance antioxidant ability, improve intestinal health, and reduce lipid deposition of seabass (*Dicentrarchus labrax*) [15], grass carp [16], and California yellowtail (*Seriola dorsalis*) [17]. However, there is no report on whether taurine can alleviate the negative effects caused by oxidized-fish-oil diets in aquatic animals. Therefore, we have carried out related research.

Channel catfish (*Ictalurus* *punctatus*), which belongs to catfish family (*Siluriformes*), is an important freshwater aquaculture fish in China. Because of its delicious meat, high nutritional value, and fast growth, it is welcomed by producers and consumers. In 2018, the production of channel catfish exceeded 390,000 tons in the world, an increase of 3.34% over the previous year [18]. Channel catfish has a high requirement for feed freshness, and feed mildew, deterioration, and oxidation will have a negative impact on growth and health. Therefore, this study aimed to investigate whether taurine can alleviate the negative effects of lipid deposition, oxidative stress, and intestinal damage induced by oxidized-fish-oil diets in juvenile channel catfish. It is of great significance to explore the side-effects of oxidized fat ingestion on the growth and health of aquatic animals for the study of fish nutrition and health, and to provide solutions for practical production and a theoretical basis for the application of taurine.

## 2. Materials and Methods

### 2.1. Preparation of Oxidized Fish Oil

Oxidized fish oil was prepared by constant temperature water bath aeration. The detailed steps are as follows: fill the beaker with fresh fish oil, place it in a constant temperature water bath at 50 °C, insert the air pump snorkel into the container and aerate it for five days. The peroxide value of the fish oil was monitored daily until it reached 897.4 meq/kg. The peroxide value of fresh fish oil was 9.2 meq/kg.

### 2.2. Experimental Diets

Three isonitrogenous and isolipid diets were designed in this experiment—fresh fish oil (FFO, control group) diet, oxidized fish oil (OFO) diet, and OFO diet with 0.2% taurine (OFOT) (Table 1). The ingredients were finely ground, sieved (0.25 mm), mixed and supplemented with fish oil and soybean oil. A 10% volume of water of the weight of the ingredients was added. After mixing, pellets were squeezed (1.0 and 1.5 mm in size) and then dried naturally in the shade. The experimental diets were then stored at −20 °C until use.

### 2.3. Experimental Animals and Feeding Experiment

Channel catfish were purchased from a fine seed farm (Wuhan, Hubei, China), and the breeding experiment was carried out in the recirculating aquaculture system of Wuhan Dabeinong Aquatic Science and Technology Co., Ltd. (Wuhan, Hubei, China). During the acclimatization period, the FFO group was fed until the channel catfish showed obvious feeding behavior, and then the fish were fasted for 24 h. Channel catfish fingerlings (average weight 6.00 ± 0.01 g) were randomly distributed into 12 breeding barrels (diameter 1.0 m, water depth 0.8 m, indoor) with three replicates in each treatment group, each containing 35 fish per replicate. During the 8-week the feeding trial, the channel catfish were manually fed three times per day (8 a.m., 12 p.m., and 5 p.m.) at 3%–5% of their body weight. The water temperature was maintained at 27.32 ± 0.17 °C, dissolved oxygen was more than 6.5 mg/L, and ammonia and nitrate were less than 0.2 mg/L.

### 2.4. Sample Collection

All experiments followed the regulations of Hunan Agricultural University for laboratory animal protection. After the experiment, growth performance was calculated after 24 h of fasting. The fish were anesthetized with MS-222 (100 mg/L, Sigma Aldrich Co. LLC., St. Louis, MO, USA) before sampling [19]. Three fish were taken from each breeding barrel for tail vein blood collection, which was collected in a 2 mL centrifuge tube and placed at 4 °C for 12 h. After that, the supernatant was centrifuged and stored at −80 °C. Three fish from each breeding barrel were quickly dissected on ice, and the liver, intestine, and skin (backside and abdomen) tissues were removed in enzyme-free centrifuge tube (1.5 mL) and put it in liquid nitrogen, then stored at −80 °C.

### 2.5. Determination of Growth Parameters

The weight gain rate (WGR), feed conversion ratio (FCR), survival rate (SR), condition factor (CF), hepatosomatic index (HSI), and viserosomatic index (VSI) were calculated, as follows:

Weight gain rate (WGR, %) = (final body weight − initial body weight)/initial body weight × 100(1)

Feed conversion rate (FCR) = total amount of the feed consumed/(final body weight-initial body weight)(2)

Survival rate (SR, %) = final number of fish/initial number of fish × 100(3)

Condition factor (CF, g/cm^3^) = 100 × whole body weight/(body length)^3^ (4)

Hepatosomatic index (HSI, %) = liver weight/whole body weight × 100(5)

Viserosomatic index (VSI, %) = visceral weight/whole body weight × 100(6)

### 2.6. Skin Pigment and Body Color Analysis

Three fish were randomly selected from each breeding barrel and tested on the backside and abdomen of each fish with a chromometer (model: 601, Beijing, China) to obtain L*, a*, and b* values. “L*” is brightness: 0–100 from black to white; “a*” is red-green: red is represented as a positive value, green is represented as a negative value; and “b*” is yellow-blue: yellow is represented as a positive value, blue is represented as a negative value. The activities of carotenoids, lutein, and tyrosinase in the backside and abdomen skin of each fish were assessed by Elisa kits (Meimian, Jiangsu, China).

### 2.7. Biochemical Index Analysis

The levels of total cholesterol (TC), triacylglycerol (TG), immunoglobulin M (IgM), complement 3 (C3), complement 4 (C4), alanine aminotransferase (ALT), and aspartate aminotransferase (AST) in serum were assayed by using a commercial kit (Nanjing Jiancheng Bioengineering Institute, Nanjing, China).

The glutathione (GSH), superoxide dismutase (SOD), glutathione peroxidase (GPx), malondialdehyde (MDA), glutathione reductase (GR), and total antioxidant capacity (T-AOC) levels in the liver were assayed by using a commercial kit (Nanjing Jiancheng Bioengineering Institute, Nanjing, China).

### 2.8. Histological Analysis

There were three replicates in each treatment group, and three fish were taken from each replicate. Liver and intestine were fixed in paraformaldehyde and embedded with paraffin wax. According to the previous experimental method, the steps of hematoxylin-eosin (H&E) staining were as follows: eight-micron tissue was taken on the glass slide with a slicer, the tissue was stained with hematoxylin, and the results were observed under an electron microscope [19]. Liver histological measurements covered 50 cells and the nuclei of the analysed tissues collected from each individual.

### 2.9. Real-Time Polymerase Chain Reaction

Total RNA from the liver and intestine was extracted using TRIzol reagent (Invitrogen, Carlsbad, CA, USA) and the quality was assessed according to Shi et al. [20]. First-strand cDNA was synthesized and RT-qPCR analysis of mRNA was performed according to a previous report [20]. The amplification efficiency was between 0.95 and 1.10, as calculated by the formula E = 10^(−1/slope)^−1. Primer sequences are shown in Table 2. With *gapdh* as the reference, the calculation is carried out according to the E = 2^−ΔΔCT^ formula [21].

### 2.10. Statistical Analysis

All data were compared by one-way analysis of variance (ANOVA), and differences between the means were tested by Duncan’s multiple-range test. All results are reported as the “mean ± S.E.”, and all statistical analyses were performed using SPSS 24.0 (New York, NY, USA). Differences were considered significant at *p* < 0.05 (*p* < 0.05) [22].

## 3. Results

### 3.1. Growth Performance

As shown in Table 3, there was no significant change (*p* > 0.05) in VSI, CF, SR, or FCR of channel catfish among treatment groups. Compared with the FFO group, WGR and final weight of the OFO group were significantly reduced, while adding taurine significantly increased (*p* < 0.05). HSI in the OFO group was significantly higher than that in the FFO group (*p* < 0.05). Compared with the OFO group, HSI in the OFOT group significantly decreased (*p* < 0.05), and there was no significant difference from the FFO group (*p* > 0.05).

### 3.2. Skin Pigment and Body Color

There was no significant change (*p* > 0.05) in L* of backside and b* of abdomen among treatment groups (Table 4). Compared with the FFO group, carotenoids of backside and L* of abdomen in the OFO group were significantly decreased (*p* < 0.05), lutein of backside and abdomen were significantly increased (*p* < 0.05). Compared with the OFO group, carotenoids, tyrosinase and a* of backside and tyrosinase and a* of abdomen in the OFOT group markedly increased (*p* < 0.05).

### 3.3. Lipid Deposition and Histological Structure of Liver

**Figure 1:**
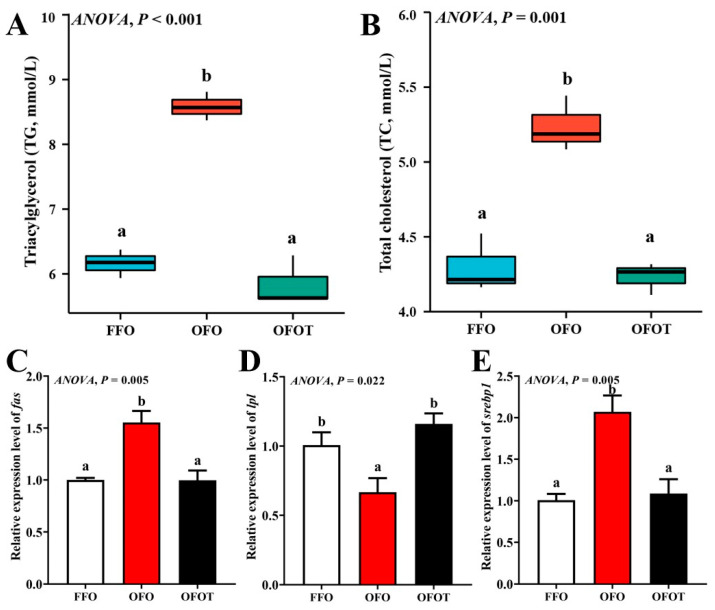
Serum biochemical indices and lipid-metabolism-related gene expression of the liver in channel catfish (*Ictalurus* *punctatus*) fed the diets. (**A**) Triacylglycerol, TG; (**B**) total cholesterol, TC; and (**C**–**E**) lipid-metabolism-related genes (*fas*, *acc,* and *srebp1*). Data indicate the mean values of three replicate cages per treatment (three fish per replicate breeding barrel). Significance was evaluated by one-way ANOVA (*p* < 0.05) followed by Duncan’s multiple range tests. Values marked with different letters are significantly different between the treatment groups.

**Figure 2:**
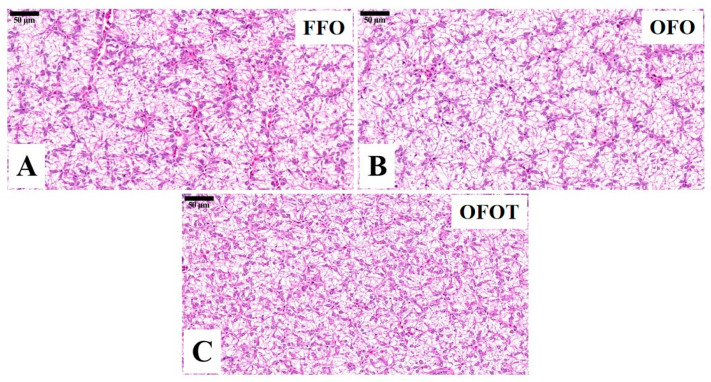
Histological characteristics of the liver in channel catfish (*Ictalurus* *punctatus*) fed the diets (H&E stain, magnification 400×). (**A**) Fresh fish oil (FFO) group; (**B**) oxidized fish oil (OFO) group; and (**C**) OFO diet with 0.2% taurine (OFOT) group.

The TG and TC contents in the OFO group were remarkably increased in comparison with the FFO group, while supplementation with 0.2% taurine significantly reduced the TG and TC contents (*p* < 0.05), there was no significant difference (*p* > 0.05) between the OFOT and FFO groups (Figure 1A,B). In comparison of the OFO and FFO groups, *fas* and *srebp1* mRNA expression in the liver of the channel catfish were significantly upregulated (*p* < 0.05), and *lpl* mRNA expression was significantly downregulated (*p* < 0.05) (Figure 1C–E). The OFOT group significantly upregulated *lpl* mRNA expression, and significantly downregulated *fas* and *srebp1* mRNA expression compared with the OFO group (*p* < 0.05). As showed in Figure 2 and Table 5, the OFO group remarkably decreased the size of nuclei, and increased the size of hepatocytes (*p* < 0.05) compared with the FFO group. The size of nuclei in the FFOT group was significantly increased compared with the OFO group, whereas the opposite result was observed for the size of hepatocytes (*p* < 0.05). Therefore, the fish fed OFO diets showed more hepatic lipid vacuolization than those fed FFO or OFOT.

### 3.4. Serum Immune Indices

The OFO group significantly reduced IgM, C4, and C3 contents, while the supplementation of taurine remarkably increased (*p* < 0.05) these immune indices compared with the OFO group (Table 6). In addition, compared with the FFO group, AST and ALT activities in the OFO group were significantly increased (*p* < 0.05), while adding 0.2% taurine remarkably decreased the activities of AST and ALT (*p* < 0.05), there was no significant difference (*p* > 0.05) between the OFOT and FFO treatment.

### 3.5. Antioxidant Indices in the Liver

**Figure 3:**
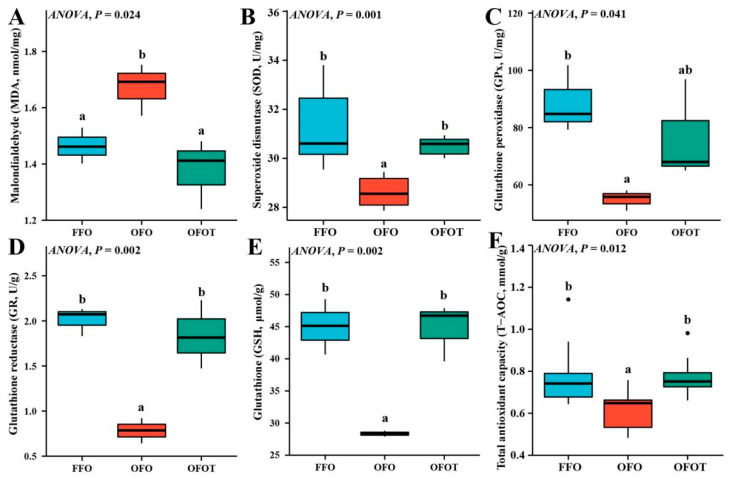
Liver antioxidant indices of channel catfish (*Ictalurus* *punctatus*) subject to different treatment. (**A**) Malondialdehyde, MDA; (**B**) Superoxide dismutase, SOD; (**C**) Glutathione peroxidase, GPx; (**D**) Glutathione reductase, GR; (**E**) Glutathione, GSH; (**F**) Total antioxidant capacity, T-AOC. Data indicate the mean values of three replicate cages per treatment (three fish per replicate breeding barrel). Significance was evaluated by one-way ANOVA (*p* < 0.05) followed by Duncan’s multiple range tests. Values marked with different letters are significantly different between the treatment groups.

As shown in Figure 3, the MDA content in the OFO group was significantly increased (*p* < 0.05) compared with that of the FFO group. The content of MDA of fish fed the 0.2% taurine supplementation diets was remarkably lower than values in fish fed OFO diets. In addition, the OFO treatment significantly decreased the levels of SOD, GPx, GR, GSH, and T-AOC, while the supplementation of taurine markedly increased (*p* < 0.05) these antioxidant indices compared with the OFO group.

### 3.6. Antioxidant-Related Gene Expression in the Liver

**Figure 4:**
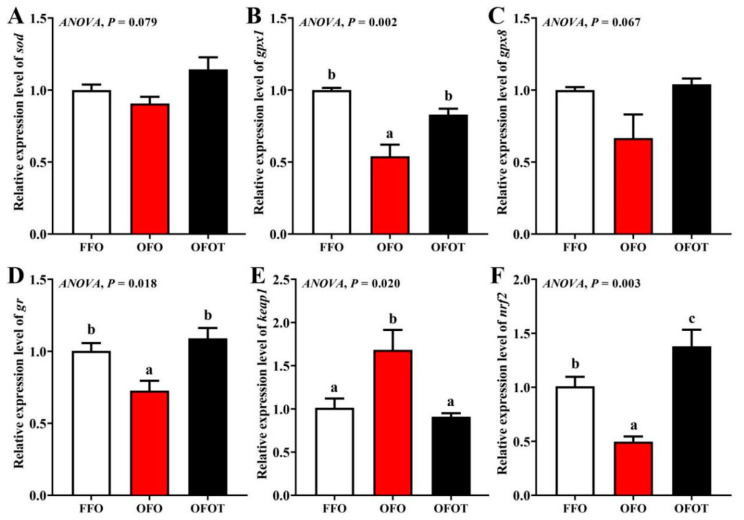
Effects of dietary taurine on liver antioxidant-related genes expression of channel catfish (*Ictalurus* *punctatus*) fed oxidized-fish-oil diets. (**A**) *sod*; (**B**) *gpx1*; (**C**) *gpx8*; (**D**) *gr*; (**E**) *keap1*; (**F**) *nrf2*. Data indicate the mean values of three replicate cages per treatment (three fish per replicate breeding barrel). Significance was evaluated by one-way ANOVA (*p* < 0.05) followed by Duncan’s multiple range tests. Values marked with different letters are significantly different between the treatment groups.

As shown in Figure 4, there was no significant difference in *sod* and *gpx8* gene expression among the treatment groups. The OFO group remarkably downregulated *gpx1*, *gr,* and *nrf2* mRNA expression, and upregulated *keap1* mRNA expression (*p* < 0.05) compared with the FFO group. *gpx1*, *gr,* and *nrf2* mRNA expression in the FFOT group were significantly upregulated compared with the OFO group, whereas the opposite result was observed for the mRNA expression level of *keap1* (*p* < 0.05)

### 3.7. Histological Structure in the Intestine

**Figure 5:**
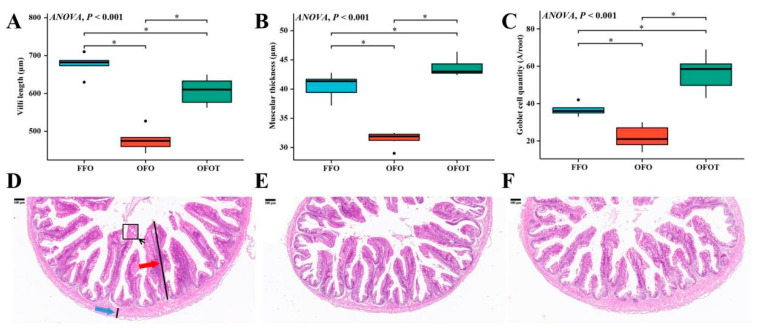
Effects of dietary taurine on intestinal morphology of channel catfish (*Ictalurus* *punctatus*) fed oxidized-fish-oil diets (magnification 40×). (**A**) Villi length; (**B**) muscular thickness; (**C**) goblet cell quantity; (**D**) FFO group; (**E**) OFO group; and (**F**) OFOT group. The red arrow indicates the villi length and the blue arrow indicates the muscular thickness, respectively. Significance was evaluated by one-way ANOVA (*p* < 0.05) followed by Duncan’s multiple range tests. * *p* < 0.05.

As shown in Figure 5, through the H&E staining analysis of the intestine, the feeding of oxidized-fish-oil diets significantly reduced the goblet cell quantity, villi length, and muscular thickness of the intestine. Compared with the OFO group, the OFOT group significantly increased the goblet cell quantity, villi length, and muscular thickness of intestine.

### 3.8. Intestinal Physical-Barrier-Related Gene Expression

**Figure 6:**
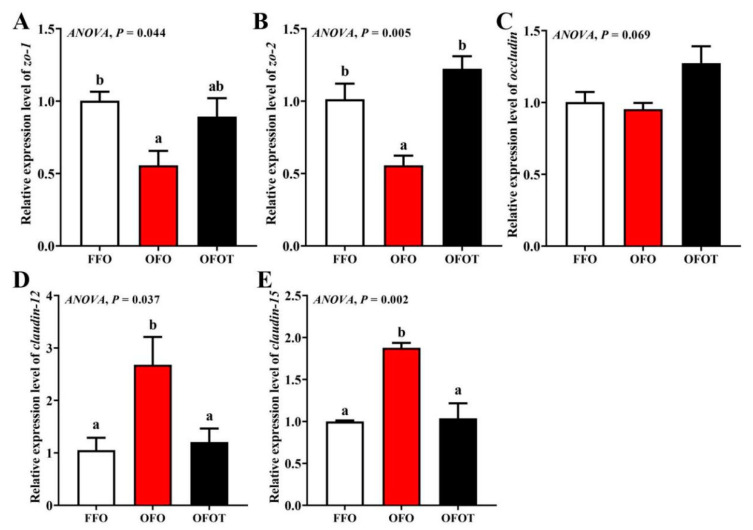
Effects of dietary taurine on intestinal physical-barrier-related genes expression of channel catfish (*Ictalurus* *punctatus*) fed oxidized-fish-oil diets. (**A**) *zo-1*; (**B**) *zo-2*; (**C**) *occludin*; (**D**) *claudin-12*; (**E**) *claudin-15*. Data indicate the mean values of three replicate cages per treatment (three fish per replicate breeding barrel). Significance was evaluated by one-way ANOVA (*p* < 0.05) followed by Duncan’s multiple range tests. Values marked with different letters are significantly different between the treatment groups.

As showed in Figure 6, there was no significant change in *occludin* mRNA expression among the treatment groups. In comparison of the OFO and FFO groups, *claudin-12* and *claudin-15* mRNA expression in the intestine of the channel catfish were markedly upregulated, and *zo-2* and *zo-1* mRNA expression were significantly downregulated (*p* < 0.05). Adding taurine remarkably upregulated *zo-1* and *zo-2* mRNA expression, and downregulated *claudin-15* and *claudin-12* mRNA expression compared with the OFO group (*p* < 0.05).

### 3.9. Intestinal Immune-Barrier-Related Gene Expression

**Figure 7:**
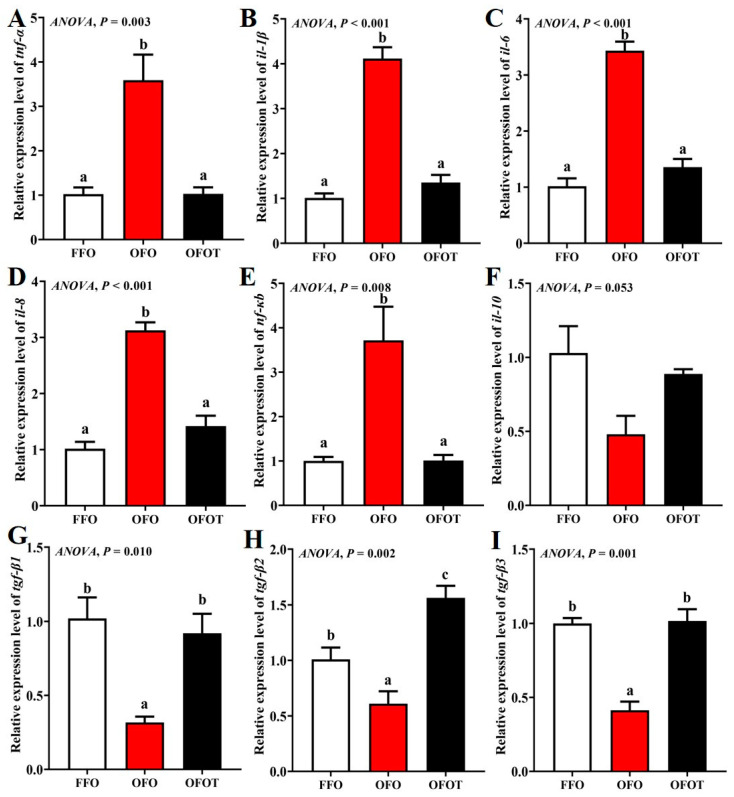
Effects of dietary taurine on intestinal immune-barrier-related genes expression of channel catfish (*Ictalurus* *punctatus*) fed oxidized-fish-oil diets. (**A**) *tnf-α*; (**B**) *il-1β*; (**C**) *il-6*; (**D**) *il-8*; (**E**) *nf-κb*; (**F**) *il-10*; (**G**) *tgf-β1*; (**H**) *tgf-β2*; (**I**) *tgf-β3*. Data indicate the mean values of three replicate cages per treatment (three fish per replicate breeding barrel). Significance was evaluated by one-way ANOVA (*p* < 0.05) followed by Duncan’s multiple range tests. Values marked with different letters are significantly different between the treatment groups.

As showed in Figure 7, the OFO group markedly downregulated *tgf-β1*, *tgf-β2*, and *tgf-β3* mRNA transcription levels, and upregulated *tnf-α*, *nf-κb*, *il-1β*, *il-6*, and *il-8* mRNA transcription levels compared with the FFO group (*p* < 0.05). *tgf-β1*, *tgf-β2*, and *tgf-β3* mRNA transcription levels in the OFOT group were markedly upregulated compared with the OFO group, whereas the opposite result was observed for *tnf-α*, *nf-κb*, *il-1β*, *il-6*, and *il-8* mRNA transcription levels (*p* < 0.05).

**Figure 8:**
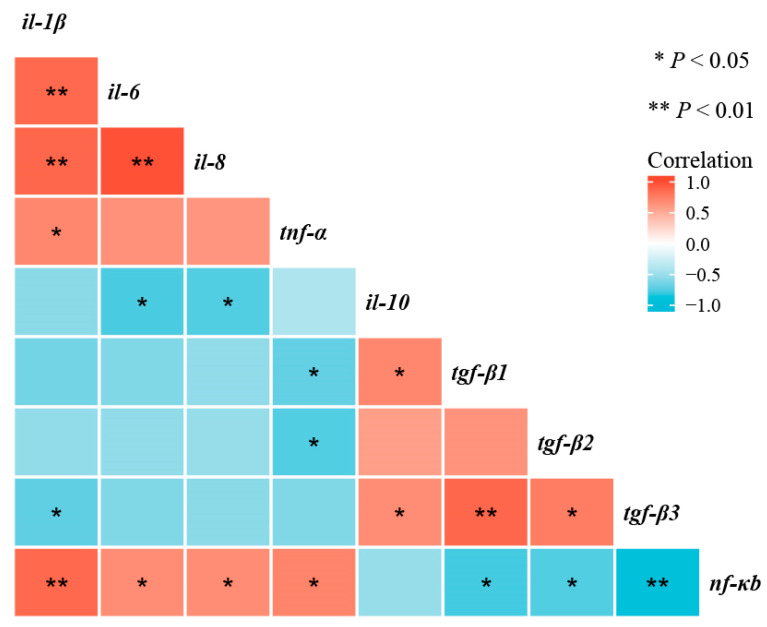
Correlative analysis of intestinal immune-barrier-related gene expression was performed using the R Programming Language. * *p* < 0.05, ** *p* < 0.01.

As shown in Figure 8, correlation analyses shown that *nf-κb* mRNA transcription level was negatively correlated with *tnf-α*, *il-1β*, *il-6*, and *il-8* mRNA transcription levels (*p* < 0.05), and positively correlated with *tgf-β1*, *tgf-β2*, and *tgf-β3* mRNA transcription levels (*p* < 0.05).

## 4. Discussion

A fresh fish oil can provide the HUFAs needed during the growth of fish [23]. HUFAs are prone to oxidative rancidity and have a negative impact on fish. In this study, oxidized-fish-oil diets significantly reduced the growth performance of channel catfish, which is similar to results obtained in juvenile hybrid grouper (♀ *Epinephelus fuscoguttatus* × ♂ *Epinephelus lanceolatus*) [24], farmed tilapia [6], orange spotted grouper [5], and yellow catfish (*Pelteobagrus fulvidraco*) [25]. One of the reasons is that toxic and harmful substances such as lipid hydroperoxides, ketones, aldehydes, and acids are produced after oxidation of fish oil, which induces oxidative stress, leads to inflammatory response, and then inhibits growth [5]. Another reason is that oxidized fish oil has reduced nutritional value compared with non-oxidized fish oil [26]. Taurine has been widely used in aquatic feeds. Adding an appropriate amount of taurine to diets can obviously increase growth performance of yellowtail kingfish (*Seriola lalandi*) [27], turbot [28], and tiger puffer (*Takifugu rubripes*) [29]. Experimental results also showed that the addition of 0.2% taurine to the oxidized-fish-oil diet obviously increased the growth performance of channel catfish, and there was no significant difference from the FFO group. There are two main reasons why taurine promotes fish growth: first, taurine has a good attractant effect [30]; second, taurine may alleviate the negative effects caused by oxidized-fish-oil diets, such as lipid deposition, oxidative damage, and inflammatory response.

Long-term feeding of oxidized-fish-oil diets can lead to the lipid deposition of liver [31]. Based on H&E staining, liver fat vacuolation is usually expressed as the size of hepatocytes and their nuclei [32,33]. In this study, oxidized-fish-oil diets led to lipid deposition in the liver of channel catfish, which was supported by the phenomenon of increased lipid vacuolization in the liver (such as smaller nuclei and larger hepatocytes), HSI, serum TC, and TG contents. Similar studies have been found in yellow catfish [25], loach (*Misgurnus anguillicaudatus*) [34] and largemouth bass (*Micropterus salmoides*) [35]. Further studies showed that oxidized-fish-oil diets resulted in liver lipid deposition due to the upregulation of the expression of lipid synthesis gene (*fas*) and the downregulation of the expression of lipolysis gene (*lpl*). Sterol-regulatory element binding protein 1 (*srebp1*) is mainly involved in the activation of enzymes related to lipid synthesis, and can promote lipid synthesis by targeting the expression of fatty-acid-catalyzing enzymes such as *fas* [36,37]. The present study showed that feeding oxidized-fish-oil diets significantly upregulated *srebp1* mRNA expression, indicating that oxidized-fish-oil diets can induce lipid deposition by regulating the mRNA expression of lipid synthesis and lipolysis. Taurine has a good function of reducing lipid deposition. It has been found in broiler chickens that taurine can reduce blood lipid content [38]. There are also studies in aquatic animals that have found that taurine can promote lipolysis of European seabass [15], white seabream (*Diplodus sargus*) [39], and Persian sturgeon (*Acipenser persicus*) [40]. In this study, the addition of taurine to oxidized-fish-oil diets remarkably reduced lipid vacuolization in the liver, HSI, serum TC, and TG contents. Furthermore, taurine remarkably downregulated the transcriptional levels of *fas* and *srebp1* in the liver, and upregulated the transcriptional level of *lpl*, indicating that taurine alleviated lipid deposition induced by oxidized-fish-oil diets. Studies have speculated that taurine has a good lipolysis effect, which may be related to the AMPK/SIRT1 signaling pathway [38]. Previous studies confirmed that activation of AMPK can inhibit the activities of FAS and ACC, thereby reducing the concentration of malonyl-CoA and enhancing CPT1 activity, thus increasing lipid catabolism and reducing lipid deposition [41,42]. However, the mechanism of taurine alleviating lipid deposition needs further study.

For animals, the oxidation diet is one of important exogenous factors leading to oxidative stress. Long-term feeding of oxidized-fish-oil diets can induce the production of reactive oxygen species in mitochondria, and excessive reactive oxygen species (ROS) can lead to tissue oxidative damage [43,44,45]. Malondialdehyde (MDA) is the final decomposition product of lipid peroxidation caused by ROS, and its content reflects the degree of peroxidation [46]. In the process of ROS removal, CAT breaks down hydrogen peroxide into oxygen and water, and SOD and GPx also play an important role, which can decrease hydrogen peroxide [47,48]. The present study showed that oxidized-fish-oil-diet feeding led to markedly a higher the content of MDA and lower the levels of CAT, SOD, GPx, GR, and T-AOC in the liver than in the FFO group. As is well known, the increase of serum AST and ALT activities is one of the important markers of liver injury [49]. Besides, the present study has showed that oxidized-fish-oil diets significantly increased serum AST and ALT activities of channel catfish, indicating that oxidized-fish-oil diets leads to oxidative stress and damage in the liver. Similar studies have found in other aquatic animals that oxidized-fish-oil diets significantly decreased antioxidant enzyme activities and increased AST and ALT activities of Wuchang bream [7] and tilapia (*Oreochromis niloticus*) [6]. Some studies have shown that taurine is a powerful antioxidant, mainly due to its stable biofilm and direct scavenging ability of ROS [50]. Furthermore, taurine can also improve antioxidant capacity by increasing the activity of antioxidant enzymes [51]. The results of this experiment also showed that the addition of taurine to the oxidized-fish-oil diet remarkably promoted CAT, GPx, GR, SOD, and T-AOC levels, whereas the opposite result was observed for the MDA level. The antioxidant capacity of taurine is related to its role as a precursor of glutathione [52], and taurine can also enhance the regeneration of glutathione from glutathione disulfide [53].

Antioxidant enzyme activity is regulated by the nrf2/keap1 signaling pathway [54]. *keap1* inhibits the expression of antioxidant genes by inhibiting the nuclear translocation of *nrf2* [55]. The present study showed that oxidized-fish-oil diets remarkably downregulated the transcriptional levels of *nrf2*, *gr*, and *gpx1* in the liver, while the transcriptional levels of *keap1* were reversed. These results were consistent with the results of antioxidant enzyme activities, indicating that long-term feeding of oxidized-fish-oil diets can reduce the antioxidant capacity of channel catfish. Previous studies in pufferfish (*Takifugu obscurus*) [56] and yellow catfish (*Pelteobagrus fulvidraco*) [57] have found that when fish are under oxidative stress, dietary taurine can increase the expression levels of antioxidant enzyme genes in the liver, thus improving antioxidant capacity. In this study, the addition of taurine to oxidized-fish-oil diets remarkably upregulated *nrf2*, *gr*, and *gpx1* mRNA expression, whereas the opposite result was observed for the transcriptional level of *keap1*. These results were consistent with the results of antioxidant enzyme activities, indicating that taurine can relieve peroxidation injury of channel catfish caused by oxidized-fish-oil diets. Similar studies have confirmed that taurine can remarkably improve antioxidant ability in juvenile turbot by regulating the *nrf2*/*keap1* signaling pathway [58]. Therefore, we speculate that taurine regulates the activity of antioxidant enzymes through the *nrf2*/*keap1* signaling pathway, thereby enhancing the ability of fish to resist oxidative stress.

Immune-active substances such as immunoglobulin and complement factor in serum play an important role in animal immune response [59]. Fish mainly rely on the non-specific immune system to respond to external environmental stimuli and pathogen invasion [60]. As a protein response system, the complement system is mainly responsible for destroying or removing pathogenic microorganisms, and is an important part of the non-specific immunity [61]. The present study showed that oxidized-fish-oil diets significantly decreased immune function, which was supported by the phenomenon of decreased serum C3, C4, and IgM contents. Some studies have indicated that dietary supplementation of taurine can alleviate acute ammonia poisoning of yellow catfish by increasing the content of total immunoglobulin in serum [57]. In addition, our previous study revealed that taurine can improve serum C3 and C4 levels in rice field eel to alleviate the immune response induced by high-fat diets [62]. The results of this study showed that the dietary supplementation of taurine to a oxidized-fish-oil diet increased serum C3, C4, and IgM contents, which indicated that taurine can improve immune function of channel catfish. Similar experimental results were found in Chinese mitten crab (*Eriocheir sinensis*) [63] and yellow catfish [64].

Further research has shown that taurine can enhance immune function though controlling intestinal inflammatory response [65]. Intestinal inflammatory response is mainly regulated by cytokines, including anti-inflammatory cytokines (including *tgf-β* and *il-10*) and pro-inflammatory cytokines (including *tnf-α il-1β*, *il-6*, and *il-8*) [66]. The present study showed that the mRNA transcription levels of *tnf-α il-1β*, *il-6*, and *il-8* were remarkably upregulated when channel catfish fed oxidized-fish-oil diets, whereas the opposite result was observed for the mRNA transcription levels of *tgf-β1*, *tgf-β2*, and *tgf-β3*. A similar study has been conducted in *Rhynchocypris lagowski*, which showed that oxidized-fish-oil diets lead to high expression of pro-inflammatory cytokines (*tnf-α*, *il-1β*, and *il-8*) and low expression of anti-inflammatory cytokines (*il-10* and *tgf-β*) [67]. Finding how to alleviate the inflammatory reaction caused by an oxidized-fish-oil diet is very important to improving the utilization rate of aquatic feed. Previous studies have reported that adding taurine significantly downregulated the expression levels of anti-inflammatory cytokines in grass carp [16] and yellow catfish [57]. The results of this experiment also shown that the addition of taurine to a oxidized-fish-oil diet dramatically downregulated *tnf-α il-1β*, *il-6*, and *il-8* mRNA expression in the liver, and upregulated *tgf-β1*, *tgf-β2*, and *tgf-β3* mRNA expression, indicating that taurine can reduce inflammatory response in the intestine induced by oxidized-fish-oil diets.

Cytokine expression in inflammatory response is regulated by various signaling pathways, among which nuclear transcription factor-κB (NF-κB), as an important signaling factor, plays an important role in inflammatory response [68]. In this study, long-term feeding of oxidized-fish-oil diets remarkably upregulated the transcriptional level of *nf-κb*. However, the addition of taurine to oxidized-fish-oil diets reversed this trend. Furthermore, correlation analyses showed that the mRNA expression level of *nf-κb* was negatively correlated with the mRNA expression levels of *tnf-α*, *il-1β*, *il-6*, and *il-8*, and positively correlated with the mRNA expression levels of *tgf-β1*, *tgf-β2*, and *tgf-β3*, which suggested that taurine inhibited the NF-κB signaling pathway to protecting oxidized fish-oil-induced inflammation response in channel catfish.

Intestinal physical-barrier function is an indispensable part of intestinal health of aquatic animals [69]. Generally speaking, the muscular thickness and villi length in the intestine are important criteria to measure the efficiency of digestion and absorption [70]. Goblet cells on intestinal villi, as typical mucous cells, play an important role in regulating the integrity of intestinal epithelial cells and the immune response to foreign antigens [71]. The present study showed that oxidized-fish-oil diets significantly reduced the villi length, goblet cell quantity, and muscular thickness of intestine. However, the addition of taurine to oxidized-fish-oil diets reversed this trend, indicating taurine can maintain the structural integrity of the intestine. An important component of the intestinal physical barrier is tight junction protein. Studies have reported that tight junction proteins are closely related to the integrity of intestinal structure, and the upregulation of transmembrane protein-related genes (including *occluding*, *zo-1,* and *zo-2*) can maintain the structural integrity of intestinal epithelial cells, while the upregulation of cytoplasmic protein-related genes (including *claudin-12* and *claudin-15*) can damage the structural integrity of intestinal epithelial cells [54,72]. This present study found that oxidized-fish-oil diets substantially downregulated intestinal *zo-1* and *zo-2* mRNA transcriptional levels of channel catfish, and upregulated *claudin-12* and *claudin-15* mRNA transcriptional levels, indicating that oxidized-fish-oil diets may increase the intestinal barrier structure damage caused by intercellular space by regulating tight junction protein genes. Taurine has been reported to enhance intestinal morphology and barrier function [73]. The results of this experiment also showed that the addition of taurine to a oxidized-fish-oil diet remarkably upregulated intestinal *zo-1* and *zo-2* mRNA transcriptional levels, and downregulated *claudin-12* and *claudin-15* mRNA transcriptional levels, indicating that taurine can repair the intestinal physical barrier damage induced by oxidized-fish-oil diets. However, the specific regulatory mechanism needs to be further studied.

## 5. Conclusions

The present study indicated that oxidized-fish-oil diets have a negative effect on growth performance, lipid metabolism, antioxidant ability, and intestinal health in channel catfish. However, addition of taurine to a oxidized-fish oil diet can increase growth performance of channel catfish. Taurine reduced lipid deposition in the liver through promoting the transcription factors of lipid metabolism including *srebp1*, *lpl,* and *fas*. In addition, our findings revealed that the supplementation of taurine alleviated oxidized fish-oil-induced oxidative damage of the liver through the Nrf2-Keap1 signaling pathway based on the transcriptional expression, and then significantly improved the activity of antioxidant enzymes. Furthermore, the current study revealed that the supplementation of taurine alleviated inflammatory response in the intestine through the NF-κB signaling pathway based on the transcriptional expression.

## Figures and Tables

**Table 1:** Composition and nutrient levels of basic diet (%, dry matter).

Ingredients	FFO	OFO	OFOT
Fish meal	10.00	10.00	10.00
Soybean meal	28.00	28.00	28.00
Rapeseed meal	20.00	20.00	20.00
Rice bran	3.00	3.00	3.00
Wheat flour	25.26	25.26	25.06
Chicken meal	9.00	9.00	9.00
Fish oil	2.00	0.00	0.00
Oxidized fish oil	0.00	2.00	2.00
Premix ^1^	1.00	1.00	1.00
Choline	0.20	0.20	0.20
Ca(H_2_PO_4_)_2_	1.50	1.50	1.50
Mold inhibitor	0.03	0.03	0.03
Antioxidants	0.01	0.01	0.01
Taurine	0.00	0.00	0.20
Approximate composition (%) ^2^			
Crude protein	36.27	36.14	36.31
Crude lipid	5.47	5.52	5.43
Crude ash	6.57	6.49	6.53
POV (meq/kg)	3.7	21.4	21.2

^1^ Provided by MGO Ter Bio-Tech (Qingdao, Shandong, China). Vitamin and mineral premix composition (mg/kg diet): KCl 200 mg, KI (1%) 60 mg, CoCl_2_·6H_2_O (1%) 50 mg, CuSO_4_·5H_2_O 30 mg, FeSO_4_·H_2_O 400 mg, ZnSO_4_·H_2_O 400 mg, MnSO_4_·H_2_O 150 mg, Na_2_SeO_3_·5H_2_O (1%) 65 mg, MgSO_4_·H_2_O 2000 mg, zeolite power 3645.85 mg, VB1 12 mg, riboflavin 12 mg, VB6 8 mg, VB12 0.05 mg, VK3 8 mg, inositol 100 mg, pantothenic acid 40 mg, niacin acid 50 mg, folic acid 5 mg, biotin 0.8 mg, VA 25 mg, VD 35 mg, VE 50 mg, VC 100 mg, ethoxyquin 150 mg, flour 2434.15 mg. ^2^ Crude protein, crude lipid, ash, and POV were measured values. The detection method was referenced to previous studies [19].

**Table 2:** Primers used for mRNA quantitative real-time PCR.

Gene	Forward Sequences (5′→3′)	Reverse Sequences (5′→3′)	Accession No.
*fas*	CTGCTGTCTGAGGGCGTAA	CGATGGCGATGAGGTTCT	NM_001200193.1
*lpl*	AGCAACATTACCCAACCTCAGC	CCAGCTACATGAGCACCCAAA	KF693235.1
*srebp1*	GTTGCGGAAGGCGATTGA	GCAGTGGGCTGTTGGGTTC	XM_017480901.1
*sod*	GACTTGGGCAAAGGTGGAAA	CACTCAGCAATGCCTATCACG	NM_001200992.1
*gpx1*	TCTGAGGCACGACCACCA	GCGTCTTTCCCGTTCACAT	NM_001200741.1
*gpx8*	TCACTTCACCGTGTTGGCTT	CCCTCAGCACTCACCAGAAA	XM_017466944.1
*gr*	GGATGTGAAGGATAAGCGAAAC	TTCGGCAACACGGGTATG	GU588318.1
*keap1*	CGGCAAGCATCTCAGTCG	TGCTCGGGTCCAACTGC	XM_017482237.1
*nrf2*	GGTCCACGCCTACCAACAA	CAGGGAGGAATGGAGGGAT	XM_017470076.1
*zo-1*	TACCAAACCGTGGATACAAACC	CTTCTATGGGTGGAGGAGGC	XM_017458510.1
*zo-2*	GAGGTCAAAGGGCAGCAAA	GAAATCTTCGGGCAGGTCA	XM_017488926.1
*claudin-12*	GCTGGGATGTTCCTCTTGATAG	AGAGCGGCGAACTCAAGG	XM_017453476.1
*claudin-15*	GTGGTTCTCGGCACATTCG	CAAGCCCTGTAGGATGAAGAAG	XM_017471911.1
*occludin*	GCATCGGTAGCGGGTCAT	GACTTGGTTGAGTTCTGCCTTG	XM_017451558.1
*tnf-α*	CGCCAGCGGTAAACACG	CCGTTGAATGTCCGAAAGG	XM_017464718.1
*il-1β*	CTGAAGGGTGGAAACAAGGAT	GGAGTCACCAGTGCCGTTT	AJ586102.1
*il-6*	GAAGATTGATACTCCGCTCCTG	GATTAAATGTAACAGCCTGGTGG	XM_017455306.1
*il-8*	TCCAAGTGCCTCCTGTTCAA	CCCTTCTTCCCTTGGACTTTAT	KP701473.1
*il-10*	GCAGGCTTACGAAAGGGTTA	CGGCGTATGAAGAACGAAGT	XM_017450800.1
*tgf-β1*	GGAACGGCTGAGTGGGTCT	TGCTTACTGAGGCGGCTATG	XM_017483625.1
*tgf-β2*	TGAAGCGGTCAGCGAATG	CTCACTCTTGTTTGGGATGATGTA	XM_017476217.1
*tgf-β3*	TCGGTGCCCTGTCCTATTG	GCGGAGAACGAGGCTTACA	XM_017476492.1
*nf-κb*	CTCAGCCCATCTACGACAACA	CGTCAGGTTCGTATCGCAGT	KF572025.1
*gapdh*	TGTCCGTTTGGAGAAGCCT	ATCAGGTCACAGACACGGTTG	NM_001201199.1

**Table 3:** Effects of dietary taurine on the growth performance of channel catfish (*Ictalurus* *punctatus*) fed oxidized-fish-oil diets.

	FFO	OFO	OFOT	*p*-Value
Initial weight (g)	6.00 ± 0.01	6.00 ± 0.01	5.99 ± 0.00	0.702
Final weight (g)	26.20 ± 0.14 ^b^	23.43 ± 0.72 ^a^	25.62 ± 0.40 ^b^	0.036
WGR	336.53 ± 1.38 ^b^	290.49 ± 11.93 ^a^	327.27 ± 6.55 ^b^	0.034
SR	97.14 ± 2.86	94.29 ± 2.86	93.33 ± 6.67	0.829
FCR	1.16 ± 0.04	1.35 ± 0.09	1.20 ± 0.08	0.223
HSI	2.26 ± 0.05 ^a^	2.74 ± 0.12 ^b^	2.46 ± 0.07 ^a^	0.003
VSI	13.20 ± 0.66	13.67 ± 0.47	13.28 ± 0.30	0.781
CF	1.52 ± 0.04	1.57 ± 0.03	1.49 ± 0.01	0.216

Note: Data indicate the mean values of three replicate cages per treatment (three fish per replicate breeding barrel). Mean values with different superscripts in a row are significantly different (one-way ANOVA, *p* < 0.05). Weight gain rate (WGR, %) = (final body weight − initial body weight)/initial body weight × 100; survival rate (SR, %) = final number of fish/initial number of fish × 100; feed conversion rate (FCR) = total amount of the feed consumed/(final body weight − initial body weight); hepatosomatic index (HSI, %) = liver weight/whole body weight × 100; viserosomatic index (VSI, %) = visceral weight/whole body weight × 100; condition factor (CF, g/cm^3^) = 100 × whole body weight/(body length)^3^.

**Table 4:** Effects of dietary taurine on skin pigment and body color of channel catfish (*Ictalurus* *punctatus*) fed oxidized-fish-oil diets.

	FFO	OFO	OFOT	*p*-Value
Backside				
Carotenoids (μg/mL)	16.00 ± 0.47 ^b^	14.04 ± 0.32 ^a^	18.42 ± 0.57 ^c^	0.002
Lutein (pg/mL)	1746.8 ± 84.5 ^a^	2331.8 ± 26.0 ^b^	2243.5 ± 17.3 ^b^	<0.001
Tyrosinase (ng/mL)	4986.3 ± 43.3 ^a^	4699.7 ± 87.4 ^a^	5583.0 ± 120.0 ^b^	0.001
L*	51.14 ± 0.49	53.24 ± 1.25	47.64 ± 2.87	0.125
a*	−5.10 ± 0.06 ^a^	−5.44 ± 0.08 ^a^	−4.54 ± 0.29 ^b^	0.008
b*	1.60 ± 0.23 ^a^	2.49 ± 0.15 ^ab^	2.07 ± 0.25 ^b^	0.031
Abdomen				
Carotenoids (μg/mL)	13.18 ± 0.23 ^a^	13.61 ± 0.07 ^ab^	14.91 ± 0.64 ^b^	0.049
Lutein (pg/mL)	2216.0 ± 21.7 ^b^	2391.8 ± 22.4 ^c^	2083.5 ± 40.1 ^a^	0.001
Tyrosinase (ng/mL)	4448.0 ± 31.8 ^a^	4309.7 ± 57.0 ^a^	4693.0 ± 75.5 ^b^	0.009
L*	82.41 ± 0.20 ^b^	81.52 ± 0.25 ^a^	81.55 ± 0.14 ^a^	0.011
a*	−3.47 ± 0.06 ^ab^	−3.55 ± 0.06 ^a^	−3.31 ± 0.03 ^b^	0.017
b*	8.19 ± 0.05	8.67 ± 0.12	8.01 ± 0.42	0.200

Note: Data indicate the mean values of three replicate cages per treatment (three fish per replicate breeding barrel). Mean values with different superscripts in a row are significantly different (one-way ANOVA, *p* < 0.05).

**Table 5:** Effects of dietary taurine on the morphometrics of the liver of channel catfish (*Ictalurus* *punctatus*) fed oxidized-fish-oil diets.

	FFO	OFO	OFOT	*p*-Value
Size of nuclei (μm)	6.57 ± 0.02	5.35 ± 0.03	6.61 ± 0.02	<0.001
Size of hepatocytes (μm)	17.03 ± 0.05	21.66 ± 0.26	17.02 ± 0.05	<0.001

Note: Data indicate the mean values of three replicate cages per treatment (three fish per replicate breeding barrel).

**Table 6:** Effects of dietary taurine on serum immune indices of channel catfish (*Ictalurus* *punctatus*) fed oxidized-fish-oil diets.

	FFO	OFO	OFOT	*p*-Value
C3 (g/L)	1.04 ± 0.03 ^c^	0.72 ± 0.02 ^a^	0.87 ± 0.03 ^b^	<0.001
C4 (g/L)	0.65 ± 0.02 ^b^	0.5 ± 0.02 ^a^	0.69 ± 0.03 ^b^	<0.001
IgM (g/L)	1.42 ± 0.08 ^b^	1.01 ± 0.03 ^a^	1.37 ± 0.07 ^b^	0.003
AST (U/L)	33.02 ± 6.31 ^a^	56.45 ± 0.97 ^b^	35.2 ± 0.42 ^a^	0.008
ALT (U/L)	8.65 ± 0.58 ^a^	13.33 ± 0.77 ^b^	7.57 ± 0.62 ^a^	<0.001

Note: Data indicate the mean values of three replicate cages per treatment (three fish per replicate breeding barrel). Mean values with different superscripts in a row are significantly different (one-way ANOVA, *p* < 0.05). C3, complement 3; C4, complement 4; IgM, immunoglobulin M; AST, aspartate aminotransferase; and ALT, alanine aminotransferase.

## Data Availability

All data generated or analyzed during this study are included in this published article.
